# Le Ventre

**Published:** 1905-03

**Authors:** 


**Le Ventre.** Par Dr. M. Bourcart et Dr. F. Cautru. I.—*Le Rein.* Pp. viii., 302. Geneva: H. Kündig. 1904.

This work consists of two parts: the first, devoted to a critical survey of the external form of the abdomen, back and trunk, and the manipulations for palpation of the various organs; the second deals with mobile or wandering kidney. The former is well illustrated in continental style, and, in particular, the effect of the corset upon the form of the trunk and abdomen is clearly demonstrated. In the second section many of the illustrations are reproduced, and the various possible causes of mobile kidney are considered. Especial importance is attached to an insufficiency of the organism "anatomo-physiologique," to defective intra-abdominal equilibrium, and to flattening of the paravertebral renal recesses. The treatment advocated for wandering kidney comprises constitutional measures, corrections of faulty dress and deportment, and, in particular, abdominal massage, the methods of employing this being fully described and illustrated. The anatomical researches are sound and the book scientific, but it chiefly deals with massage as applied to the treatment of nephroptosis and enteroptosis in general.

